# Supplementary material for: Applying the COM-B behaviour model to understand factors which impact 15–16 year old students’ ability to protect themselves against acquirement of Human Papilloma virus (HPV) in Northern Ireland, UK
Source: PLOS Glob Public Health. 2024 Apr 17;4(4):e0003100. doi: 10.1371/journal.pgph.0003100 (PMC11023437; doi:10.1371/journal.pgph.0003100)
Supplement: S1 Appendix — (DOCX) [file pgph.0003100.s001.docx]

**S1 Appendix:**

**Focus group questions asked to participants in alignment with the COM-B model and TDF domains**

**Capability**

| Psychological capability | Knowledge | Do you feel that you have enough knowledge to protect yourself from acquiring HPV ?  What knowledge is required to protect you from acquiring HPV? |
| --- | --- | --- |
| Psychological capability | Memory, attention and decision processes | What aspects of a HPV educational intervention are important to aid your retention of HPV information and facilitate decision making regarding HPV? |
| Psychological capability | Psychological skills | Do you feel that you have the psychological skills to make decisions about all aspects of HPV?   - Prompts: Maturity; Consent |

**Opportunity**

| Social opportunity | Social influence | What external social factors influence your access to information about HPV and the HPV vaccination? |
| --- | --- | --- |
| Physical opportunity | Environment/Resources | What external physical factors influence your access to information about HPV? |

**Motivation**

| Reflective motivation | Social/Professional Role and Identity | To what extent do you perceive HPV and associated HPV education to be relevant to you as an individual? |
| --- | --- | --- |
| Reflective Motivation | Optimism | To what extent are you confident that HPV education could be successfully implemented? |
| Reflective Motivation | Belief about consequences | What do you think could happen if you received HPV education at this age? |
| Automatic Motivation | Reinforcement | To what extent are there any incentives for you to protect yourself against acquiring HPV? |
| Automatic Motivation | Emotion | What emotions drive your decisions regarding HPV vaccination, education and screening? |
