# Supplementary material for: Applying the COM-B behaviour model to understand factors which impact 15–16 year old students’ ability to protect themselves against acquirement of Human Papilloma virus (HPV) in Northern Ireland, UK
Source: PLOS Glob Public Health. 2024 Apr 17;4(4):e0003100. doi: 10.1371/journal.pgph.0003100 (PMC11023437; doi:10.1371/journal.pgph.0003100)
Supplement: Attachment — Submitted filename: Response to reviewers.docx [file pgph.0003100.s003.docx]

Response Table (6^th^ February 2024)

|  | Reviewer |  |
| --- | --- | --- |
| The role of the church in sexual education seems to be absent from this article. It would be beneficial to include some background information on this aspect, as one would anticipate that both parents and students in such religious settings would be influenced by the church’s stance.  Are Protestant and Catholics different (refer: demographics)? – clarification needed | 1 and 2 | Thank you for highlighting this omission of background. The authors have now added a concise background to the introduction section to explain the historical divisions between the Catholic and Protestant religions in Northern Ireland. |
| Narrate a brief summary about your qualitative study design | 2 | Additional information has been added to the beginning of the methods section as recommended. |
| Could you provide additional details on the planning and execution of the focus groups? Was there consistency in group composition across sessions, or did the same participants engage in every discussion within their respective groups? | 1 | Table 1 provides all of the details of the group composition.  More details of the planning and execution of the focus groups has been added to the end of the methods section. |
| In certain groups, you have exclusively Catholic participants, while others comprise a mix of religious beliefs. Do you believe that this variation could influence the outcomes of your study? | 1 | This has now been addressed in the ‘Opportunity’ section of the discussion. |
| Could the church play a role in raising awareness about this issue? It might be worthwhile to consider adding this perspective to the “Actions Needed to Support Behavior Change” section of the article. This could provide a comprehensive view on how religious institutions can contribute to the discourse and promote positive change. | 1 | A section has been added as recommended at the end of the Opportunity section in the discussion. |
| Figure 1 labelling is incorrect | 2 | The figure has been altered so that the information is now correct. |
| The headings in the discussion have not been numbered correctly. | 2 | Numbers have been added as recommended. |
| Please add year to Gotvall et al. in the discussion section | 2 | The year has been added as recommended. |
| Statement highlighted in the conclusion section should be removed as they can not consent legally until 18 years old | 2 | Apologies for not making this clearer, but students in the UK (including Northern Ireland) can legally self-consent at 16 years old and between 12-15 years if they are found to be Gillick component.  We have added a statement highlighting this in the discussion.  We felt, in agreement with the editor, that the conclusion can remain as originally presented. |
| There are lots of inconsistencies in the style of referencing | 2 | The referencing has been modified to Vancouver style. Elsevier will edit any further possible inconsistencies in the referencing as part of the printing process, based on previous experience with this system of submission. |

-
