# Supplementary material for: Applying the COM-B behaviour model to understand factors which impact 15–16 year old students’ ability to protect themselves against acquirement of Human Papilloma virus (HPV) in Northern Ireland, UK
Source: PLOS Glob Public Health. 2024 Apr 17;4(4):e0003100. doi: 10.1371/journal.pgph.0003100 (PMC11023437; doi:10.1371/journal.pgph.0003100)
Supplement: Attachment — Submitted filename: Response Table March 2024.docx [file pgph.0003100.s005.docx]

Response Table

| Reviewer Comment | Reviewer | Response |
| --- | --- | --- |
| References need to be in a consistent format | Reviewer 2 | The authors have used PLOS ONE Public Health guidelines which adheres to The International Committee of Medical Journal Editors (ICMJE). See link  <https://www.nlm.nih.gov/bsd/uniform_requirements.html>  The standard sample journal reference given is  Halpern SD, Ubel PA, Caplan AL. Solid-organ transplantation in HIV-infected patients. N Engl J Med. 2002 Jul 25;347(4):284-7.  It also requests the author to list the first six authors, followed by et al. The authors have now aligned to this format.  The authors have also aligned to the website references with the ICMJE guidelines also. Example taken from these guidelines for part of a homepage/Web site is as follows:  American Medical Association [Internet]. Chicago: The Association; c1995-2016 [cited 2016 Dec 27]. Office of International Medicine; [about 2 screens]. Available from: <https://www.ama-assn.org/about/office-international-medicine> |
| Figures should be removed from the manuscript | Editor | The figure has been removed as recommended. |
